# Development of a Universal Prompt as a Scalable Generative AI-Assisted Tool for USMLE Step 1 Style Multiple-Choice Question Refinement in Medical Education

**DOI:** 10.1007/s40670-025-02334-7

**Published:** 2025-02-25

**Authors:** Youngjin Cho, Grace L. Park, Gabi N. Waite, Abhijay Mudigonda, John L. Szarek

**Affiliations:** 1 Department of Medical Education, Geisinger Commonwealth School of Medicine, Geisinger College of Health Sciences, Scranton, PA USA; 2 Department of Biomedical and Translational Sciences, Carle Illinois College of Medicine, University of Illinois Urbana-Champaign, Urbana, IL USA; 3 Geisinger Commonwealth School of Medicine, Geisinger College of Health Sciences, Scranton, PA USA

**Keywords:** Multiple-choice questions, Generative AI, Medical students, Prompts for medical education, Assessment writing

## Abstract

We developed a generative artificial intelligence (genAI)-assisted tool enabling learners to receive feedback on, revise, and clone multiple-choice questions aligned with learning objectives. Initially designed as a custom GPT, we adapted it to a universal prompt for platform-agnostic, equitable access. This innovation exemplifies readily adaptable genAI-enhanced learning driven by pedagogy.

**Supplementary Information:**

The online version contains supplementary material available at 10.1007/s40670-025-02334-7.

The process of creating multiple-choice questions (MCQs) encourages students to engage with the content, apply knowledge, and understand the alignment between learning objectives and assessment, improving their test-taking strategies [1]. Despite these benefits, students often struggle to progress beyond the initial writing stage due to the lack of feedback on improving the quality of their MCQs. Existing generative artificial intelligence (genAI)-based MCQ tools focus on producing practice questions, often requiring paid subscriptions with instructions hidden. Our innovation fills this gap by creating a universal *platform-agnostic* prompt for genAI to assist students with question critique and revision steps with embedded bias and patient-centered language check, which is often lacking in other genAI MCQ tools. Our approach enhances learning and simultaneously addresses equity and access issues in technology use.

At Geisinger Commonwealth School of Medicine, pre-clerkship students write MCQs in small groups in medical science courses. They are given an item-writing guide and a question template (Supplementary Information, SI, 1) with a pre-assigned topic. The questions are shared as peer-reviewed study resources without faculty review, which leads to inconsistent quality and underutilization by students.

In response, we created a tool that leverages common chatbots to provide formative feedback on student-created MCQs. Our design process was grounded on the education principles that (1) timely feedback improves performance; the tool should give constructive feedback for iterative improvement; (2) assessment is driven by learning objectives; the tool must explicitly check for learning objective alignment; and (3) all learners must have equitable access to education; the tool should mitigate disparities caused by financial or technological barriers. The process was also guided by genAI principles that AI is used to address a meaningful educational outcome and enhance the experience of learners and educators in an ethical manner.

The prototype was a custom GPT (Open AI), which offered critiques on MCQs, revision, and alternative questions (see SI 2 for development process). Because students could not access the custom GPT without a paid subscription, our interim solution was for faculty to input all questions into the GPT and share the output with students. A single faculty member was able to return over 330 questions to the students, with positive feedback supporting scalability and increased engagement in MCQ exercises (SI 2). While this ensured equal access, it increased faculty workload and raised potential compliance issues, with some institutions requiring a specific privacy-controlled chatbot.

To overcome these limitations, we developed a more scalable and accessible solution: a universal prompt (Table 1) that can be used with any chatbot, by transferring essential components of the custom GPT into a single prompt (SI 2). All instructions needed to critique questions are contained within the prompt without requiring a separate knowledge file or memory, thereby lowering the threshold for use for a genAI beginner. This prompt was evaluated by four faculty from two medical schools, with varying MCQ revision experiences, and a student with no chatbot use history, using free versions of three chatbots (ChatGPT 4o, Claude Sonnet 3.5, and CoPilot). They confirmed the consistent functionality of the prompt while recognizing variations in tones and styles of each chatbot. This eliminates the financial and access barriers to using the genAI tool while maintaining the following features to enhance students’ MCQ writing learning experience.

1. Comprehensive Critiques: The prompt guides users through a systematic critique covering all essential elements of MCQ development, including learning objectives, vignettes, answer choices, and explanations.
2. USMLE Alignment: The prompt ensures adherence to NBME item writing guidelines [2].
3. Image Handling: Images used in question (e.g., X-ray) can be separately uploaded onto the chatbot, depending on the capacities of the chatbot.
4. Educational Best Practices: It incorporates additional best practices, including patient-centered language use, bias checks, and focus on knowledge application rather than recall.
5. Iterative Improvement: The prompt offers revision and optional alternative “cloned” questions, promoting continuous improvement of students’ question-writing skills.

**Table 1:** Universal prompt for MCQ feedback, revision, and cloning

You are a specialized tutor for assisting students in creating and refining medical MCQs, with a focus on USMLE Step 1 style. You will provide structured critiques for each MCQ component, ensuring factual accuracy and adherence to high-quality USMLE question standards. The students will submit the question. Then, you follow this process
1. MCQ Critique and Generation Process: use the following structure to generate and critique each MCQ:
a) Learning Objectives: Check if learning objectives are provided. Assesses alignment between learning objectives, discipline, and the question's testing point b) Clinical Vignette: Write the case as a single narrative paragraph without providing each part separately
• Include the following components in a logical sequence: Patient demographics (age, gender identity if relevant); Site of care; Chief concern (presenting symptoms and duration); Relevant patient history (past medical, family, psychosocial); Physical examination findings (including vital signs); Results of diagnostic studies (if applicable); Initial treatment and subsequent findings (if applicable) • Use "man/woman/boy/girl" rather than "male/female" unless the distinction is clinically relevant • Avoid using first names or fictitious names • Refer to patients by their age and gender (e.g., "A 45-year-old woman") • Uses "reports" or "states" instead of "complains" when describing patient symptoms
c) Question Stem: Formulate a clear, focused, and closed lead-in question. Ask for the BEST answer, not one that is TRUE/FALSE. Ensure it can be answered without seeing the options ("cover-the-options" rule) d) Answer Choices:
• Provide four to five plausible answer options • Ensure relevance to the stem, grammatical consistency, homogeneity, and plausibility • Check for balance in length and content • Identify and avoid technical flaws such as absolute terms, grammatical cues, or convergence
e) Explanation:
• Identify and explain the correct answer • Explain why it's the most appropriate answer based on evidence-based guidelines or expert consensus. Briefly explain why the other answer options are less correct or incorrect
f) Overall Assessment:
• Provide a concise assessment of whether the question is good (not requiring a lot of revision) or needs work
g) Language and Sensitivity:
• Ensure all medical terminology is up-to-date and racially and culturally sensitive • Use patient-centered language and avoid stigmatizing descriptions • Consistently use "chief concern" instead of "chief complaint."
h) Application of Knowledge:
• Assess whether the question tests application of knowledge rather than recall of isolated facts.
2. Key Points for Vignette Construction: Present information in a logical sequence as outlined above. Include only relevant information needed to answer the question. Avoid excessive "red herrings" or irrelevant information that could confuse test-takers. Use precise language and avoid vague terms. Focus on common or potentially catastrophic problems; avoid rare conditions unless specifically testing on them.
3. Revision and Alternatives:
• Be critical of the question in giving the critique on the structure of the question, the factual accuracy, grammatical correctness, and relevance of the clinical scenario to the question and answers • After providing a critique, ask, "Would you like to see a revision incorporating these suggestions?" Hold providing revision, until the user answers • If yes, provide a revised version of the question and explanations • If no (or after providing the revision), ask, "Would you like to see two alternative questions on the same learning objectives, clinical presentation, and discipline?" • If yes, provide two alternative questions that are of equal or greater difficulty than the original. Use a different clinical scenario and make the correct answer different from the original • For both the revision and alternatives, adhere to USMLE guidelines and provide step-by-step explanations and rationale 4. Continuous Improvement: You note and correct grammatical and factual errors in all parts of the question Are you ready for the user to submit the question?

The entire prompt is shown. The prompt enables feedback customization through additional instructions during critique (e.g., focus on different Bloom’s taxonomy levels rather than application) without requiring structural changes. Learning objectives are established during question generation/cloning, not during the critiquing and revision process. The prompt can be modified to review alignment with other outcomes or course context, such as organ systems, rather than learning objectives or discipline. The function of parts of the prompt and detailed guidance on customization options that were built into the design of the prompt are detailed in SI 5

Instructions on how to use the prompt and variations of the workflow are provided (SI 3): Educators can use the published case vignette-based MCQ drafting prompt [3] to generate a draft of questions, and then use our universal prompt in tandem to revise and clone them. This allows the rapid building of a bank of practice questions aligned with teaching material that faculty can vet. The prompt can also be used as an instruction to create a custom GPT, allowing users to skip the step to copy the prompt each time they submit a question (SI 4).

Faculty and students can use any chatbot to improve their questions and create additional questions. As users input the prompt and review the critiques, they develop a deeper understanding of effective question construction, potentially improving overall assessment quality. The output includes a wide range of knowledge and case scenarios, allowing users to overcome the lack of local expertise. It mitigates the additional financial burden on students paying for external resources for practice questions. In support, all evaluators noted the ease of use, the ability to review questions critically, and the value of the step-by-step critiques. The student evaluator endorsed that peer students with limited genAI experience and chatbot access would greatly benefit from it.

Common pitfalls of genAI, such as bias, inaccuracy, incomplete understanding, and inconsistent output, will occur as with any other genAI use. Faculty and students need to be grounded in basic genAI literacy and exercise rigorous vetting and cross-review of the outputs.

This innovation equips medical students with a tool to critique and create MCQs grounded in pedagogical principles, independent of resource limitations or institutional constraints. The equitable outcome stems from transforming a successful custom GPT into a universal prompt. Future studies are planned to quantitatively measure the improvement of question-writing skills and examine the tool’s impact on academic performance.

## Supplementary Information

Below is the link to the electronic supplementary material.

Supplementary file1 (DOCX 25 KB)

Supplementary file2 (DOCX 1575 KB)

Supplementary file3 (DOCX 34 KB)

Supplementary file4 (DOCX 132 KB)

Supplementary file5 (DOCX 1233 KB)

## Data Availability

Data supporting the findings reported in the article is available from the corresponding author upon reasonable request.

## References

[1] Shah MP, Lin BR, Lee M, Kahn D, Hernandez E. Student-written multiple-choice questions-a practical and educational approach. Med Sci Educ. 2019;29(1):41–3. 10.1007/s40670-018-00646-5. 34457447 10.1007/s40670-018-00646-5 PMC8368101

[2] NBME® item-writing guide: learn how to write questions that more effectively assess your students’ knowledge. Downloadable at https://www.nbme.org/educators/item-writing-guide, Accessed 7 Nov 2024.

[3] Kiyak YS, Kononowicz AA. Case-based MCQ generator: a custom ChatGPT based on published prompts in the literature for automatic item generation. Med Teach. 2024;46(8):1018–20. 10.1080/0142159X.2024.2314723. 38340312 10.1080/0142159X.2024.2314723

