# Supplementary material for: Development of a Universal Prompt as a Scalable Generative AI-Assisted Tool for USMLE Step 1 Style Multiple-Choice Question Refinement in Medical Education
Source: Med Sci Educ. 2025 Feb 25;35(2):611–3. doi: 10.1007/s40670-025-02334-7 (PMC12058601; doi:10.1007/s40670-025-02334-7)
Supplement: Supplementary file 1 — Supplementary file1 (DOCX 25 KB) [file 40670_2025_2334_MOESM1_ESM.docx]

**Cho et al.,** Development of a Universal Prompt as a Scalable Generative AI-Assisted Tool for USMLE Step 1 Style Multiple-Choice Question Refinement in Medical Education

**Supplementary Information 1. Instruction on writing MCQ and question submission template provided to the students**

1. **Writing Multiple Choice Questions**

**Introduction**

In order to ensure efficient generation of high-quality assessment questions, we drafted this brief guide to writing single best answer multiple choice questions (MCQs). Writing good MCQs is a skill that develops over time, but this brief review will help your group write good questions for use in retrieval practice.

The single best answer format tests knowledge and comprehension, but also allows

for assessment of higher-level functions such as application analysis, synthesis and

evaluation. Each question comprises a few lines/paragraphs of text explaining the clinical scenario (‘the stem’), a single line stating the question itself (‘the lead-in’), and a list of up to five options (one correct answer and four distractors). Each of these

components is discussed below.

**The stem**

Clinical scenarios are conceived simply as the background to the knowledge or

ability to be tested. The information that the stem contains should be complete, concise, clear and unambiguous, omitting any extraneous details. Write the text of the stem in a consistent tense (present or past).

The stem must include:

- the age and sex of the patient;
- if relevant, the patient’s domicile or occupation;
- if relevant, the setting of care (e.g. outpatient clinic, accident and emergency department);
- the presenting chief concerns and its duration
- relevant medical, family and social history;
- relevant drug treatment;
- relevant physical examination findings; and
- results of relevant investigations

Questions should each have a stem comprising no more than five sentences (six

lines) of text and no more than six investigation results. For reference values of laboratory tests, refer to the NBME Table of reference Laboratory values which is attached. Avoid abbreviations, jargon, or terms that may not be understood. Use generic names for all drugs.

**The lead-in**

Present the question as a single dilemma and pose it in such a way that the test taker

can anticipate the five options (the ‘cover test’ allows you to check this).

Recommended formats include:

- What is the most likely cause/diagnosis/explanation?
- What is the most appropriate investigation/treatment/next step in management?

**Avoid** negatively worded questions, such as:

- What is the least likely diagnosis?
- Which of the following is not involved in the pathophysiology?

Negatively worded questions are not only likely to be misread by test takers who are

expecting to identify the most correct answer but also present an artificial challenge that has no equivalent in everyday clinical practice.

**The five options**

Make sure that all the options are:

- relevant to the stem and follow grammatically from it;
- related to the question; and
- balanced in length and content and not overcomplicated.

List the options in alphabetical order and label them A to E. The four distractors

should be closely related to the preferred option but less correct. They must nevertheless be both plausible and realistic. Do not use “All of the Above” or “None of the Above” as options

Finally, remember to indicate the correct answer.

**Explanations to questions**

For each question, please provide an explanation for the answers indicating why the right answer is correct and the others are wrong (less correct).

**Terminology recommendations**

Instead of … use ….

- alcohol abuse ... alcohol use disorder
- substance abuse ... substance use disorder
- tobacco abuse ... tobacco use disorder (or tobacco use)
- elderly ... older adult
- compliant ... adherent
- noncompliant ... non-adherent
- obese patient ... patient with obesity
- homeless patient ... patient experiencing homelessness
- complaint….concern

**Constructing good quality questions: Summary (checklist)**

1. Identify the point you wish to test based on the objectives provided for the topic. This helps to ensure that the point is aligned with the objectives. Try to limit the question to a single objective on which the lead-in can be focused.
2. Identify four alternatives to the correct answer, which will act as plausible distractors. Make sure that these are either incorrect, or indisputably less correct than the correct answer.
3. Build the stem as a fictional scenario that includes all the information necessary to select the correct answer (and no more).
4. Check that the stem is written as economically as possible, and that the lead-in and the alternative answers follow logically from it.
5. Prepare the explanation which should include why the correct answer is correct and the wrong answers are wrong.
6. **Question Submission Template**

Team Number:

Clinical Presentation (assigned):

Discipline (assigned):

Pre-work title (select from available prework):

Learning objectives (select from learning objectives included in the prework):

Question Stem and Lead-in:

Answer Choices:

Answer: Correct answer alphabet - text

Explanations:

- Correct answer is correct because:
- Incorrect answers are incorrect because:
