# Supplementary material for: Development of a Universal Prompt as a Scalable Generative AI-Assisted Tool for USMLE Step 1 Style Multiple-Choice Question Refinement in Medical Education
Source: Med Sci Educ. 2025 Feb 25;35(2):611–3. doi: 10.1007/s40670-025-02334-7 (PMC12058601; doi:10.1007/s40670-025-02334-7)
Supplement: Supplementary file 2 — Supplementary file2 (DOCX 1575 KB) [file 40670_2025_2334_MOESM2_ESM.docx]

**Cho, et al.,** Development of a Universal Prompt as a Scalable Generative AI-Assisted Tool for USMLE Step 1 Style Multiple-Choice Question Refinement in Medical Education

**Supplementary Information 2. Process of GPT instruction and prompt development and testing**

The custom GPT and universal prompt process involved countless rounds of iterative processes with multiple testing and adjustments based on outputs and index questions. Thus, we describe the general flow of the process as the detailed records of each testing and adjustment process are hard to capture and beyond the scope of the article.

**Step 1. Defining the desired goal and users**

We grounded initial design processes on principles of ethical and responsible AI use in education. In particular, we focused on the principles that genAI is used to address a meaningful educational outcome or challenge, enhance the experience of both learners and educators, augment human intelligence, not to replace, and mitigate harmful bias and equity issues [1],

In addressing meaningful educational outcomes, we first identified the gap in providing meaningful feedback to students' question writing, then determined AI's main desired outcomes/output as providing structured feedback on the submitted MCQ. We then framed the desired AI intent and orientation as "Instructor Proxy facing Students," meaning that AI faces learners on behalf of the instructor [2] and should provide a pedagogically supported critique of students' input as if they would receive it from the instructor. This intent and orientation were aligned with our educational goal of students using the custom GPT or prompt to receive critique.

**Step 2. Crafting the initial GPT instruction**

The initial prompt to be used as an instruction in the custom GPT was crafted using a simple RTF framework (Role/Personal; Task; Format/Output) where we specified the Role to be a tutor as instructor assistant, Task to provide critique, revise and clone, and Format to be following the question writing guide provided to the students (Supplementary Information 1) and NBME item writing guide for USMLE Step 1 style. Our primary emphasis of the initial prompt draft was to transfer the structured critiquing process of faculty as experienced item writers into logical and defined steps in the prompt.

**Step 3. Review, Refine, and Iterate of the GPT instruction**

We established index questions for output review and refinement of the GPT instruction and prompts. Those are student-written questions with a specific flaw(s), features, or varying qualities that served as a good or poor example. Index questions’ flaws or features matched the specific components of the MCQ to be critiqued and tested; for example, missing learning objectives, with negatively phrased lead-in, insensitive terminology such as “red man syndrome” for vancomycin infusion syndrome, or those with graphs and radiological or histological images.

The additional steps of adjusting for common misconceptions, providing examples, selecting knowledge files for custom GPT, and testing for outputs generally followed the approaches provided by others [3, 4].

Evaluation of outputs generated by custom GPT or the universal prompts to guide the refinement process followed the RACCA Framework, which stands for Relevance, Accuracy, Completeness, Clarity, Coherence, and Appropriateness [5, 6]. The RACCA framework can apply to both the refinement of the prompt or the evaluation of the AI output. How the RACCA framework applies to the evaluation of the output is described further:

- Relevance: The output directly addresses the intended tasks (critique, revision, and cloning)
- Accuracy: The output contains information with precise terminology, is correct, and with minimal ambiguity
- Completeness: Output contains all necessary context, and details are produced – critiques all components of the MCQ question as prompted
- Clarity: Straightforward and simple language, clear for students to understand
- Coherence: Logical flow that each part of outputs builds on the previous one, following the prompt
- Appropriateness: Tone and content are suitable for the medical students

Based on the evaluation of output, the instruction of the custom GPT was iteratively refined. For example, we noted that giving simple instructions to evaluate "patient-centered language" was often not enough, and the example of "replacing chief complaint with chief concern" was necessary within the prompt.

We evaluated the output of close to 150 student-submitted questions with the custom GPT of which instruction was iteratively refined based on the output. This was in addition to numerous tests and refinements of the prompt through index questions, as described above.

As the outcome of the custom GPT development, the MD1 class of 118 students received detailed feedback on 112 questions, 112 revisions, and 224 cloned questions across four MCQ writing exercises scheduled within a semester (a total of 336 returned to the students). A sole faculty member could complete critique, revision, and two clones of each of the 28 submitted MCQ questions in each MCQ writing exercise in a couple of hours, often limited by the daily maximum of the chat allowed. Informal feedback from the students supported the increase in awareness of learning objectives, confidence and utilization of peer-written questions, and the general question construction process. Faculty observed that student question quality improved with each exercise. This supported the scalability and learning enhancement potential of the GPT.

**Step 4. Transfer of the custom GPT instruction into a universal prompt**

The general process of constructing and refining a prompt is the same as refining the instruction of custom GPT, as described above. The main focus of the universal prompt was to 1) eliminate the use of knowledge files in the custom GPT and 2) produce a concise, clear, and stepwise prompt that can be easily understood not only by the chatbot but also by the users to provide the educational value of reading the prompt to learn how to critique MCQ appropriately.

As an example of the knowledge file elimination, we determined that simply prompting it to avoid "convergence" was enough for most platforms to follow the instructions rather than including several examples as a knowledge file. We also determined that most chatbots did not require several examples of a USMLE-style question as a knowledge file but needed a succinct summary of the clinical vignette summary within the prompt.

We utilized both GPT builder and Claude Sonnet 3.5 to refine the prompt. We provided the prompt draft, the output examples, and the desired ideal output and asked each tool to suggest how to improve the universal prompt. We then tested the suggestion and repeated the review, refine, and iterate process.

**Step 5. Cross-platform testing of universal prompt**

To validate the universal prompt's effectiveness, we tested it across three different AI chatbot platforms using 20 student group-authored questions that had previously undergone critique with our custom GPT. These questions assessed foundational science objectives spanning cardiovascular, respiratory, renal, and musculoskeletal dermatology systems. Our evaluation focused primarily on comparing the relevance and completeness of outputs to those produced by the custom GPT. Following Collins, Black, and Rarey's approach [6], we developed a standardized checklist to systematically assess the universal prompt's functionality across the three chatbots.

The resulting performance validation table below presents a cross-platform comparison across ChatGPT 4o, Claude 3.5 Sonnet, and Microsoft 365 Copilot. The evaluation metrics were structured in five rows, examining completion rates for critique steps, revision capabilities, cloning functions, frequent output variations, and features missed during critique. Both ChatGPT 4o and Claude 3.5 Sonnet achieved 95% completion (19/20) for critique steps, while Microsoft 365 Copilot reached 90% (18/20). All three platforms demonstrated perfect completion rates (20/20) for both revision and cloning tasks. Each platform exhibited distinct behavioral patterns: ChatGPT 4o tended to use numerical labeling (7/20), Claude 3.5 Sonnet typically waited for user confirmation (6/20), and Microsoft 365 Copilot often provided automatic revisions and cloning without being asked by users (8/20).

As with any chatbot use, there were occasional inconsistencies in the output, which again emphasizes the notion that the users need to check each output and have a basic understanding that additional input as in-context correction may be needed for any chatbot. Examples of features missed by platforms included knowledge application checks, with Microsoft 365 Copilot occasionally missing the instruction to critique language usage around gender and “complaint” and pointing out unnecessary details in the vignette. These missed critiques were often automatically corrected in revision, and if not, a quick prompt pointing to the omission produced an adequate response. Users and faculty may encounter similar variations and missed features, needing to “tell” the chatbot to fix the omission.

**TABLE1. Performance validation: Cross-platform comparison of completion rates**

| Performance Metric | ChatGPT 4o | Claude 3.5 Sonnet (“normal” style) | Microsoft 365  Copilot |
| --- | --- | --- | --- |
| Critique steps (a-h) completion | 95% (19/20) | 95% (19/20) | 90% (18/20) |
| Revision completion | 100% (20/20) | 100% (20/20) | 100% (20/20) |
| Cloning completion | 100% (20/20) | 100% (20/20) | 100% (20/20) |
| Frequent output variation | Numerical labeling of steps 35% (7/20) | Waited for user confirmation before providing detailed explanations in cloned questions 30% (6/20) | Automatic revision/cloning without user asking 40% (8/20) |
| Example of features missed in critique* | Evaluating application of knowledge (1/20) | Evaluating application of knowledge (1/20) | Evaluating application of knowledge (2/20)  Language: male/female, complaint (3/20)  Unnecessary details (2/20) |

*typically corrected in revision without mentioning it or with a prompt pointing to omission/error

We observed that the depth and style of feedback varied across platforms, with Microsoft 365 Copilot typically providing more succinct responses compared to ChatGPT 4o and Claude 3.5 Sonnet. An example of a question and corresponding Copilot critique are shown below.

The data suggests that **the universal prompt maintains its effectiveness across different platforms**, with only minor variations in performance and presentation style. The high completion rates for core functions (revision and cloning) indicate that the prompt is robust and platform-agnostic, achieving its goal of being a universal tool for MCQ refinement.

**FIGURE 1.** Example of the student submitted question and the corresponding critique using the universal prompt with Copilot. Screen captures of the interaction are shown.


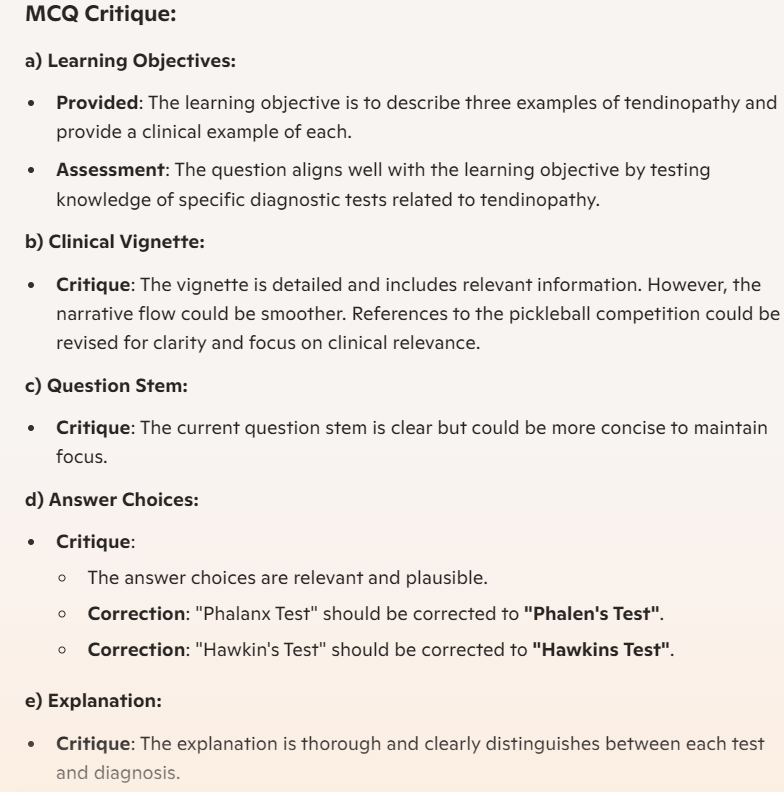


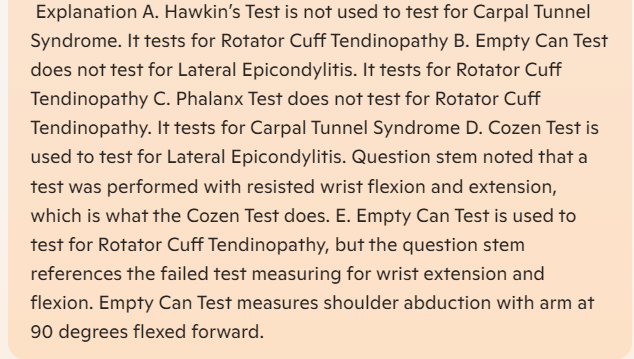

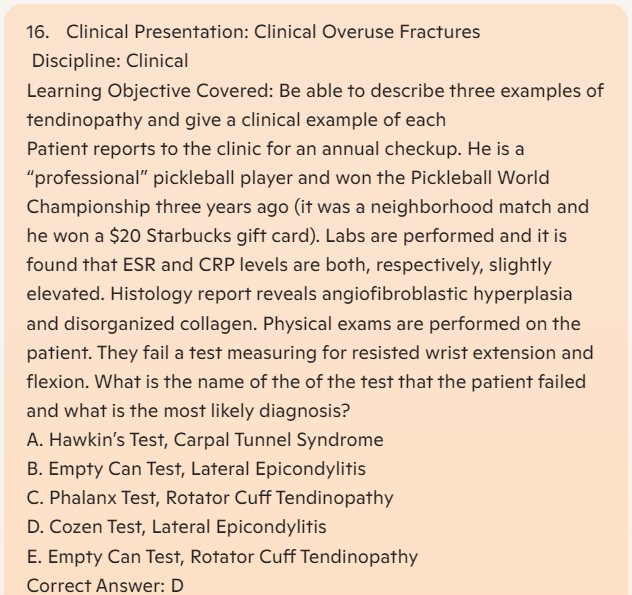


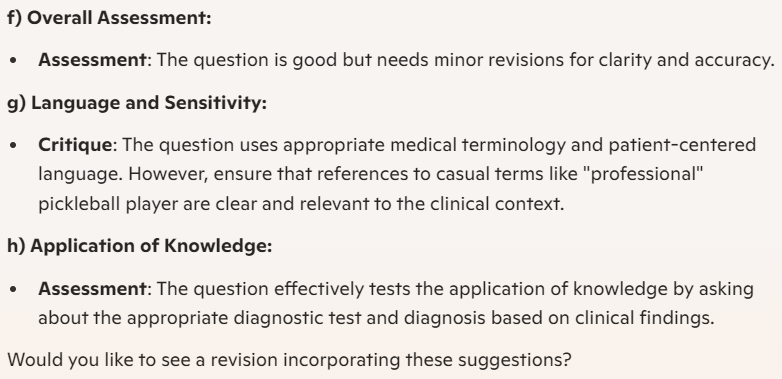


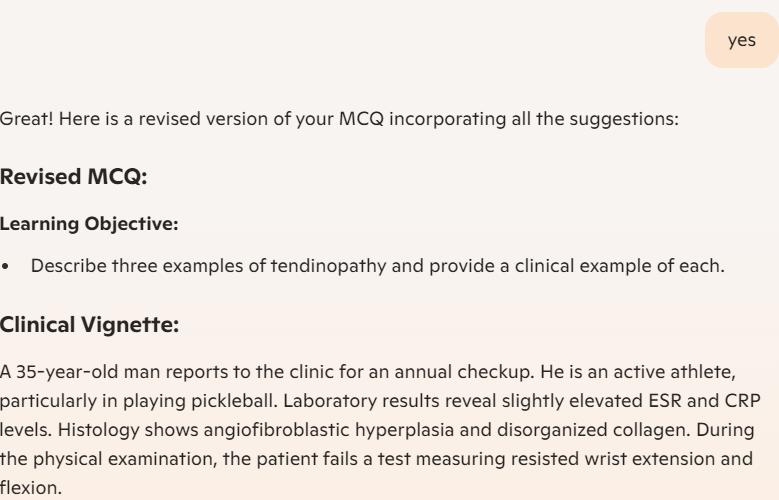


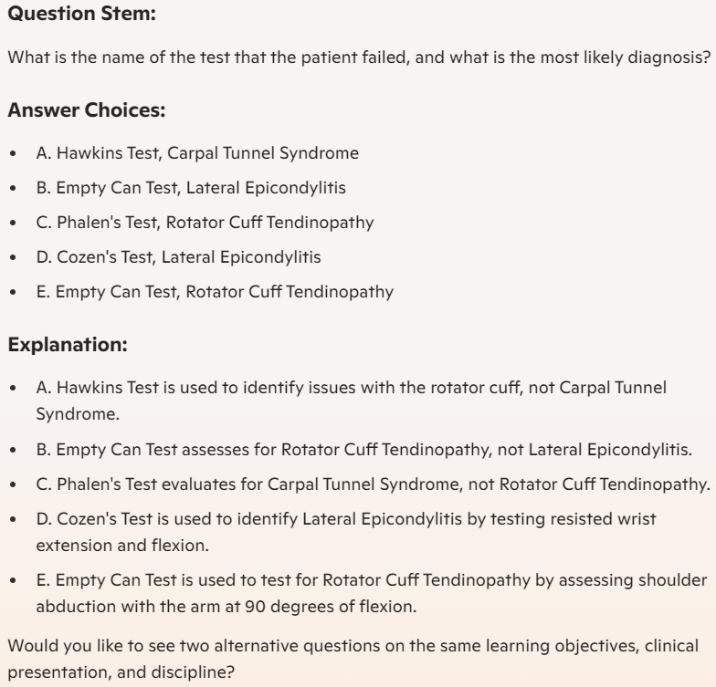


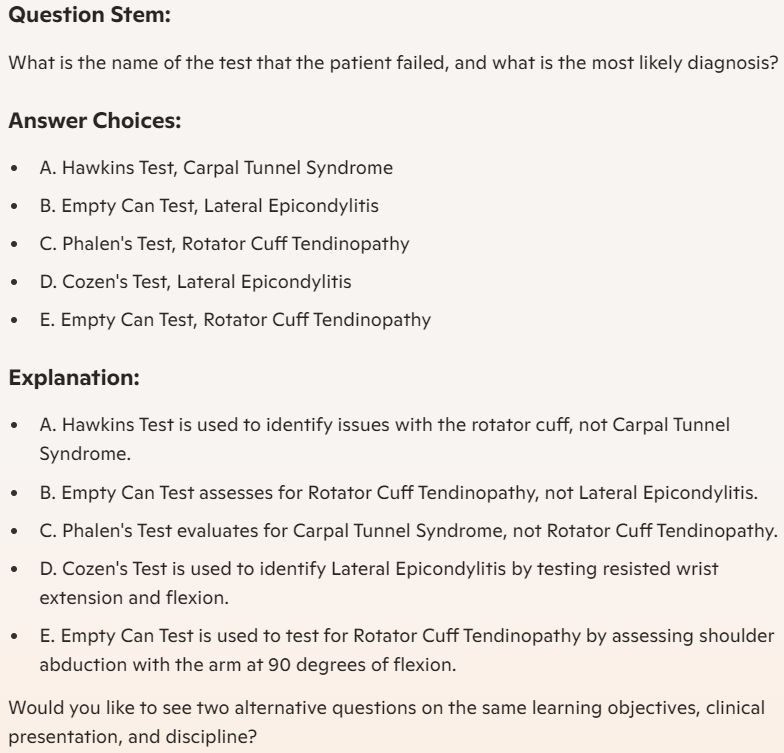


**REFERENCES**

1. American Medical Association, Advancing AI in medical education through ethics, evidence and equity. Sep 2023. <https://www.ama-assn.org/practice-management/digital/advancing-ai-medical-education-through-ethics-evidence-and-equity>
2. Pike at al., Framing generative AI in education with the GenAI intent and orientation model. EDUCASE 2024. <https://er.educause.edu/articles/2024/6/framing-generative-ai-in-education-with-the-genai-intent-and-orientation-model>
3. Mollick, Ethan R. and Mollick, Lilach, Instructors as Innovators: a Future-focused Approach to New AI Learning Opportunities, With Prompts. The Wharton School Research Paper 2024, Available at SSRN: https://ssrn.com/abstract=4802463 or <http://dx.doi.org/10.2139/ssrn.4802463>
4. Masters K, Benjamin J, Agrawal A, MacNeill H, Pillow MT, Mehta N. Twelve tips on creating and using custom GPTs to enhance health professions education. Med Teach. 2024 46(6):752-756. doi: 10.1080/0142159X.2024.2305365. Epub 2024 Jan 29. PMID: 38285894.
5. Hobson JK. Demystifying Prompt Engineering: Unveiling the Art Behind Effective AI Communication. LinkedIn. 2024. <https://www.linkedin.com/pulse/demystifying-prompt-engineering-unveiling-art-behind-effective-20t5c/>
6. Wadsworth R. Frameworks for Effective Prompting: Using the RACCCA and CLEAR Models. LinkedIn. 2024. <https://www.linkedin.com/pulse/frameworks-effective-prompting-using-raccca-clear-models-wadsworth-sjpke/>
7. Collins BR, Black EW, Rarey KE. Introducing AnatomyGPT: A customized artificial intelligence application for anatomical sciences education. Clin Anat. 2024 Sep;37(6):661-669. <https://doi.org/10.1002/ca.24178>. Epub 2024 May 9. PMID: 38721869.
