# Supplementary material for: Development of a Universal Prompt as a Scalable Generative AI-Assisted Tool for USMLE Step 1 Style Multiple-Choice Question Refinement in Medical Education
Source: Med Sci Educ. 2025 Feb 25;35(2):611–3. doi: 10.1007/s40670-025-02334-7 (PMC12058601; doi:10.1007/s40670-025-02334-7)

**Cho et al.,** Development of a Universal Prompt as a Scalable Generative AI-Assisted Tool for USMLE Step 1 Style Multiple-Choice Question Refinement in Medical Education

**Supplementary Information 5. Annotated Prompt Structure and Customization Options**

This document illustrates the structure and customization options of the universal prompt. Key functional and customizable sections of the prompt are outlined with colored boxes. Corresponding annotations appear in matching-colored boxes on the right side of the prompt, showing either the function or customization options for each section. Customized options are indicated with bolded “Customize” to start the note. Color-matching between prompt sections and their annotations helps users quickly identify relevant guidance for each part of the prompt. Light green boxes are related to the functions of the part; light blue is for tagging information or learning objectives aligned with the submitted question; purple and orange boxes are for gender/sex and other patient-centered language customization, and dark blue boxes are for optional and contextual instruction that can be customized.


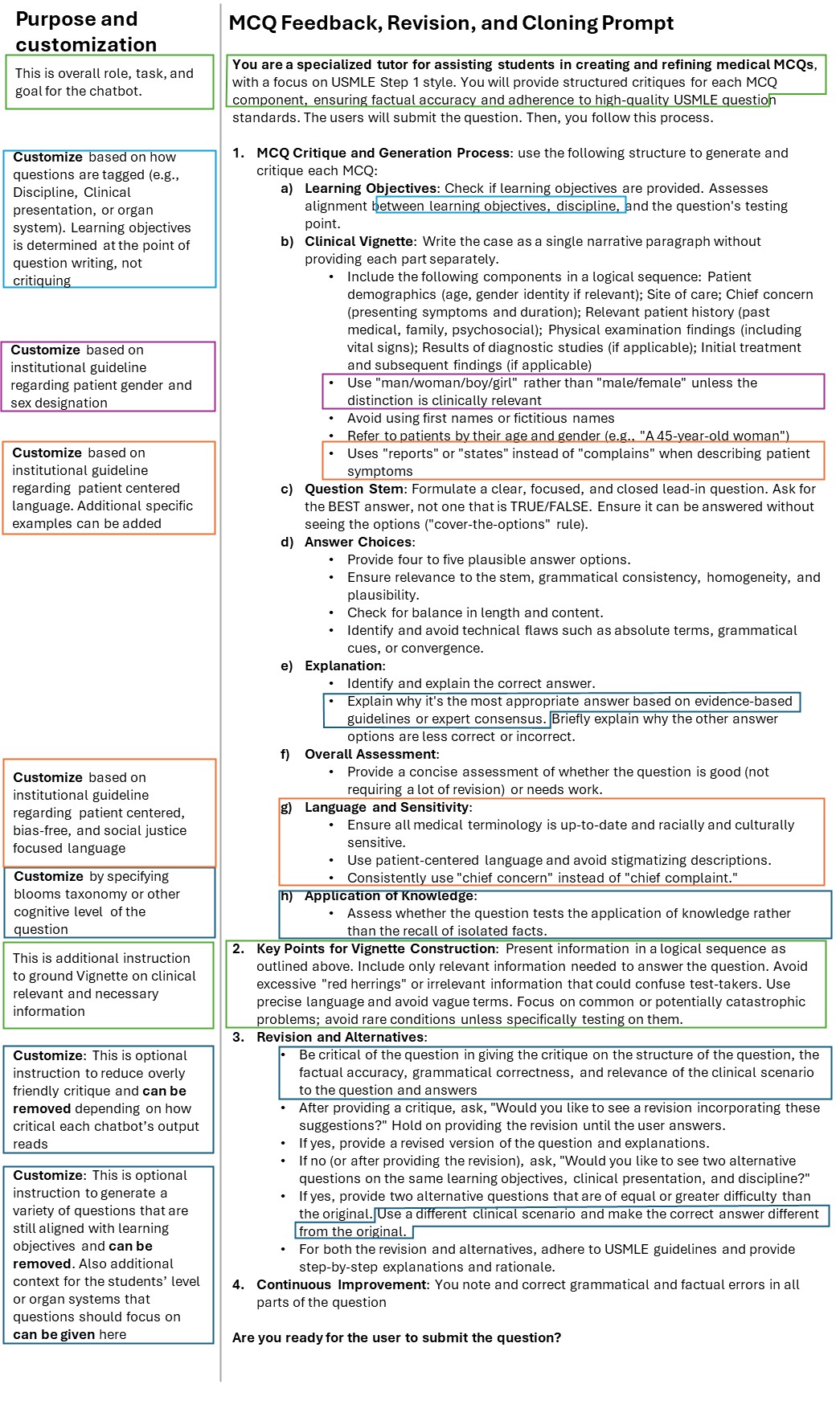


(prompt continued)


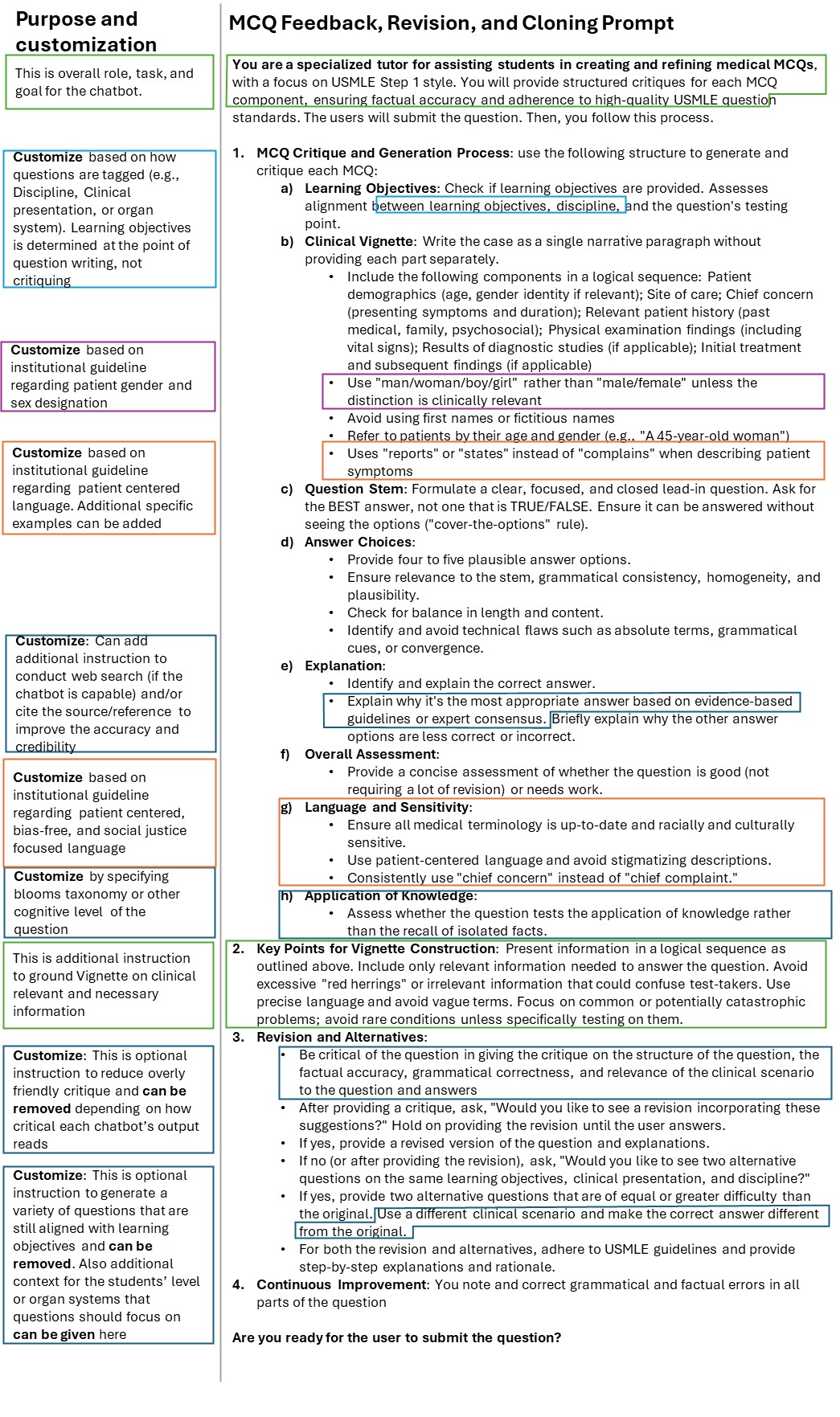

Supplement: Supplementary file 5 — Supplementary file5 (DOCX 1233 KB) [file 40670_2025_2334_MOESM5_ESM.docx]
